# The Role of Neutrophil Extracellular Traps in Hepatocellular Carcinoma. What Are the Implications of Anesthetic Techniques? A Narrative Review

**DOI:** 10.3390/ijms27010155

**Published:** 2025-12-23

**Authors:** Sergiu Sargarovschi, Alexandru Leonard Alexa, Oszkar-Karoly Bondar, Daniela Ionescu

**Affiliations:** 1 Department of Anesthesia and Intensive Care I, “Iuliu Hatieganu” University of Medicine and Pharmacy, 400012 Cluj-Napoca, Romania; 2 Association for Research in Anesthesia and Intensive Care (ACATI), 400162 Cluj-Napoca, Romania; 3 Romania Onco-Anaesthesia Research Group, ESAIC, 1000 Brussels, Belgium; 4 Outcome Research Consortium, Cleveland, OH 44195, USA

**Keywords:** NETs, hepatocellular carcinoma, anesthesia

## Abstract

Neutrophil extracellular traps (NETs)—webs of DNA and granular proteins expelled by neutrophils—have been implicated in hepatocellular carcinoma (HCC) progression. NETs promote tumor angiogenesis, facilitate invasion/metastasis, and enable immune evasion. Recent data suggest that perioperative factors, including anesthetic techniques, may modulate NET formation (NETosis), thus potentially influencing oncologic outcomes. We conducted a literature review of experimental and clinical studies on NETosis pathophysiology and involvement in HCC and how anesthetic techniques may modulate NET formation and, implicitly, cancer outcomes. NET biomarkers such as citrullinated histone H3 (CitH3), cell-free DNA (cfDNA), and myeloperoxidase–DNA complexes (MPO-DNA) are elevated in HCC patients and correlate with tumor spread, showing diagnostic and prognostic potential. Perioperative anesthetic choices may influence NET activity and immune function. Regional anesthesia and local anesthetics (e.g., lidocaine infusion) attenuate the surgical stress response and preserve anti-tumor immunity. Notably, lidocaine may modulate NET formation and, in a few studies published so far, was shown to reduce postoperative NET markers and other pro-metastatic factors (MMP-9, VEGF) in cancer surgery. In conclusion, NETosis is a process that is strongly implicated in HCC biology. Data published so far suggest that the clinical significance of NETosis may lie in its potential as a marker for disease evaluation and progression, including during the perioperative period. Preliminary results suggest that lidocaine may have a role in decreasing NETosis. Future large randomized trials are needed to exactly quantify these effects. Targeting NETs may be another way to influence HCC outcomes.

## 1. Introduction

HCC is the most frequent form of liver cancer, accounting for 80% of all liver cancers and for 290,000 deaths in 2020 [1]. It is estimated that these numbers will increase over time [1]. Approximately 30% of HCC patients undergo surgery, and this percentage will increase in the future following the increase in the incidence of HCC [2,3].

Therefore, an increasing number of patients will require surgery and, implicitly, anesthesia, not only due to the increased incidence of HCC but also due to the development of minimally invasive techniques in liver surgery [2].

On the other hand, there are debates in the literature on the potential influence of anesthetic agents on postoperative short- and long-term outcomes in cancer patients, raising concerns about opioid use and increasing interest in intravenous lidocaine as an alternative [4]. In the last few years, there have been studies showing that lidocaine may have anti-tumor effects, in which DNA demethylation, interactions with mRNAs, the direct inhibition of tumor cell proliferation and migration, Src and NfkB inhibition, and antiangiogenic effects are involved [5,6]. In a prospective clinical trial, Alexa et al. reported that, in the lidocaine group, the incidence of recurrence 1 year after curative resection in colorectal cancer was significantly reduced [7]. A very recent post hoc analysis in lung cancer surgical patients found improved OS with a non-significant trend for improved DFS in the intravenous lidocaine group as compared with those given a placebo or lidocaine paravertebrally [8]. In the case of opioids, the evidence remains mixed; in a retrospective analysis of cancer outcomes in patients receiving opioids, the results were inconclusive [9,10]. Moreover, a nationwide prospective cohort study assessing short-term perioperative opioid use suggested that it may not significantly influence cancer recurrence [11].

One of the most recently described mechanisms that may contribute to cancer biology is NETosis. For this reason, a narrative review on the role of NETosis in HCC oncogenesis and its potential modulation by anesthetic agents would be of interest. To our knowledge, such a review has not been published before.

For a better understanding of the role of NETosis in HCC biology, some details regarding the etiology and pathophysiological mechanisms are necessary. HCC usually develops through a pathologic liver parenchyma—mostly chronic liver disease or liver cirrhosis [12].

Risk factors for HCC include hepatitis B (HBV) and C (HVC) virus infections, chronic alcoholic hepatitis, nonalcoholic fatty liver disease, aflatoxin B1-contaminated food, and a wide variety of metabolic (e.g., diabetes), nutritional, and dietary factors [13]. Additionally, metabolic dysfunction-associated fatty liver disease (formerly NAFLD) has emerged as a significant risk factor for HCC in the absence of advanced fibrosis, likely mediated by lipotoxicity, oxidative stress, and insulin resistance [14,15].

As the main pathogenetic mechanisms, chronic HBV and HCV induce HCC through host–viral interaction with the subsequent activation of a T-cell immune response, leading to repeated necrosis–inflammation–fibrosis cycles, the induction of oxidative stress, and host DNA methylation alterations [15,16,17]. In the case of chronic HCV-induced HCC, the capacity of chronic HCV to evade the host immune response may be added [17]. The inflammatory response is elicited by damage-associated molecular patterns (DAMPs), released from damaged or necrotic cells, and pathogen-associated molecular patterns (PAMPs), released by viruses via the activation of certain receptors, like Toll-like receptor (TLR), C-type lectin receptors, and others [18].

In the case of alcohol-induced HCC, inflammation plays an important role, generating the release of inflammatory cytokines (TNFα, interleukin-1β, IL-6), cycles of necrosis–inflammation–regeneration, and oxidative stress, leading ultimately to cirrhosis and HCC. Aflatoxin may generate HCC via genetic mutations [17].

Other common features in HCC pathogenesis are genomic instability and genetic events such as the inactivation/mutation of tumor suppressor p53, the overexpression of β-catenin, and phosphorylated signal transducer and activator of transcription 3 (pSTAT3), phosphorylated extracellular signal-regulated kinase 1/2 (pERK1/2) and spleen tyrosine kinase (S) SYK (S) expression [17,19,20].

Additionally, the tumor microenvironment (TME) plays a crucial role in HCC development and progression. The cellular component of the HCC TME comprises helper and immune cells and the extracellular matrix (ECM) [21,22]. Immune cells are represented by tumor-associated neutrophils (TANs), tumor-associated macrophages (TAMs), regulatory T cells (Tregs), inhibitory B cells, myeloid-derived suppressor cells (MDSCs), cytotoxic T cells (CD8+ T), dendritic cells (DCs), and natural killer (NK) cells. Helper cells are represented by cancer-associated fibroblasts (CAFs), hepatic stellate cells (HSCs), and vascular endothelial cells [21,22,23]. The cellular component and the non-cellular component form an immunosuppressive TME that promotes HCC development [21]. The TME is also associated with therapeutic options such as immune or targeted therapy [21,22,23].

Given the rising incidence of HCC, this review examines the role of inflammation and immune dysregulation from the perspective of NET involvement in hepatocarcinogenesis. We summarize the current clinical evidence regarding the influence of anesthesia on NETosis and explore how anesthetic techniques may modulate perioperative inflammation and, ultimately, HCC outcomes. To provide the necessary context, we first outline the biology of neutrophils, inflammatory signaling, and the mechanisms driving NET generation, together with their relevance in HCC progression. Building on this foundation, the review discusses the effects of anesthetic agents on NETosis, their impacts on tumor biology, and the potential implications for postoperative prognosis in patients with HCC.

## 2. Literature Review Design

A literature search was conducted on NETs and HCC using multiple databases, including PubMed, The Cochrane Library, Web of Science, Scopus, and Google Scholar.

For this review, we used the following keywords to identify relevant articles: HCC, anesthesia, propofol, opioids, lidocaine, local anesthetics, dexmedetomidine, NETosis, cancer, and inflammation. From a total of 1027 articles, we rejected all unrelated articles and included 81 in our review. Most of the studies were preclinical or conducted in animal models, while the clinical research involved only small patient cohorts.

## 3. Neutrophils and the Role of Inflammation in HCC Biology

Neutrophils are one of the most important contributors to acute and chronic inflammatory responses, are activated in response to infections, and are involved in the clearance of pathogens and in sterile inflammation triggered by damage-associated molecular patterns (DAMPs) [24,25,26].

In addition to their traditional functions, neutrophils are also involved in HCC pathogenesis, depending on their phenotype, and play a central role in tumorigenesis, metastasis, and local tumor progression [27,28,29]. Two main phenotypes of TANs have been described, N1 and N2, with N1 displaying an anti-tumor profile while N2 TANs have pro-tumor properties [27].

N2 TANs promote tumorigenesis by expressing increased levels of neutrophil elastase (NE), cathepsin G, arginase, and other pro-tumor factors [30,31]. Other mechanisms by which neutrophils may be involved in tumorigenesis include the enhancement of tumor cell survival, invasiveness and metastasis, extracellular matrix remodeling, and angiogenesis [27,30,32]. Contrary to the N2 phenotype, the N1 phenotype of TANs has anti-tumor effects by increasing the production of reactive oxygen species (ROS), the activation of the innate and adaptive immune responses, and anti-inflammatory effects via microRNA-223 transmission to macrophages [30,33,34].

Recent data show that neutrophils may have a much larger spectrum of pro-tumor and anti-tumor functions depending on the TME [30,35]. These include pro-inflammatory pro- and anti-tumor and anti-inflammatory pro- and anti-tumor actions, respectively [36]. Moreover, recent studies show that the neutrophil-to-lymphocyte ratio (NLR) not only reflects immune activity in the TME but also mirrors the interplay between innate and adaptive immunity in cancer patients [37]. The increase in neutrophil numbers leads to an increase in matrix metalloproteinase-9 (MMP-9) levels, which appears to contribute to angiogenesis and tumor progression, leading to a poor prognosis [38]. A study using a transgenic mouse model (RIP1-Tag2) raised the hypothesis that neutrophils themselves may be the source of MMP-9 [39].

A particular type of cells that deserves mentioning is polymorphonuclear myeloid-derived suppressor cells (PMN-MDSCs), which have the same origin and undergo the same differentiation as neutrophils but have differences in the expression of surface markers [40].

These cells are immunosuppressive: they directly inhibit T cells and may also inhibit NK cells. MDSCs produce high levels of ROS, while having MPO and MMP expression [40]. PMN-MDSCs are important in clinical practice as prognostic factors for tumor progression and metastasis [40,41,42].

The manipulation of different neutrophils or their functions may render tumor cells or the microenvironment more susceptible to immunotherapies or they may constitute therapeutic targets [31].

## 4. Neutrophil Extracellular Trap Formation

NETs are structures composed of DNA, antimicrobial proteins, and histones. They are released into the extracellular space by neutrophils, activated by bacterial stimuli, inflammation, or other triggers [43,44]. Although NETosis has long been recognized as a mechanism of host defense against infections, its involvement in autoimmune diseases, metabolic disorders, and cancer pathogenesis is currently under investigation [45]. NET formation is influenced by external stimuli acting on neutrophils, as well as by intercellular interactions mediated through cytokines, chemokines, and other signaling pathways [43].

Pro-inflammatory cytokines such as IL-1β, IL-8, GM-CSF, IFN-γ, IL-6, and IL-17A have been shown to trigger or amplify NETosis; these cytokines promote neutrophil recruitment, activation, and survival and protein arginine deiminase 4 (PAD4) upregulation, thereby facilitating chromatin decondensation and NET release [45]. Additional factors contributing to NET formation are chemokine ligands (CCLs), such as CXCL1, which, alone, may not directly trigger NETosis, but they prime neutrophils to respond more vigorously to secondary stimuli like ROS production, PAMPs, or platelet–neutrophil interactions [46]. CXCL5, which intensifies the clustering of neutrophils at inflammatory sites, creates a high-density environment that favors NETosis [46]. Nonetheless, by blocking CXCL8-CXCR2 expression, cancer progression was delayed in preclinical studies [47]. Another factor implicated in lytic NETosis is DEK, a nuclear chromatin-binding protein that mediates chromatin decondensation in a manner similar to MPO [43,48].

### 4.1. Types of NET Formation

#### 4.1.1. Lytic (Suicidal) NET Formation

This involves the disintegration of the nuclear envelope, chromatin decondensation, and the release of DNA–histone complexes [49].

##### NADPH Oxidase-Dependent NET Formation

This process is mediated by nicotinamide adenine dinucleotide phosphate (NADPH) oxidase-dependent pathways, along with Raf-Mek-Erk, protein kinase C pathways, and reactive oxygen species (ROS) production [50]. ROS generated by NADPH oxidase promote the activation and release of neutrophil elastase (NE) from azurophilic granules, which partially depends on myeloperoxidase (MPO), into the cytosol, where NE together with MPO can translocate to the nucleus to initiate histone degradation and chromatin decondensation [51,52].

##### NADPH Oxidase-Independent NET Formation

This process is triggered by fungal ionophores (A23187, ionomycin, nigericin), which induce calcium influx into neutrophils and activates the PAD4 enzyme [50,53]. PAD4 activation leads to the citrullination of histones and other proteins [53]. Citrullinated histones lose part of their positive charge, weakening their interactions with negatively charged DNA [54]. This observation raises the hypothesis that histone citrullination promotes chromatin decondensation [50,53]. A further characteristic of Ca^2+^ ionophore-induced NETosis is the production of mitochondrial ROS (mROS) through the activation of the small-conductance calcium-activated potassium channel SK3 [55]. There are still many unanswered questions regarding whether NETosis can occur in the absence of ROS activity [53].

#### 4.1.2. Vital NET Formation

This is a non-lytic process where neutrophils remain functional after releasing NETs. It can be induced rapidly (within minutes) and is independent of cell death [53]. This mechanism was described by Pilsczek et al. in response to Staphylococcus aureus. It is NADPH-independent and involves the packaging of chromatin into vesicles, allowing the neutrophil to remain alive despite the loss of a functional nucleus [53,56]. Another proposed mechanism is the involvement of mitochondrial DNA in the NETosis process [53].

## 5. Implications of NETs in HCC Biology

There are a number of processes implicated in HCC biology, in which NETs may intervene and are actively involved. NETs actively shape the HCC microenvironment to favor tumor growth and angiogenesis [57]. These NET-derived factors directly stimulate cancer cell proliferation. For example, NE and high-mobility group box 1 protein (HMGB1) released during NETosis can drive tumor cell cycling and growth [58]. NE can enter tumor cells and degrade tumor suppressors or remodel the extracellular matrix, while HMGB1 (a damage-associated molecular pattern) engages receptors like RAGE/TLR4 on hepatoma cells to activate NF-κB signaling, activating a pro-survival, pro-proliferation program [58,59]. In HCC models, NETs, internalized by tumor cells, trigger a TLR4/9-mediated inflammatory cascade, upregulating cyclooxygenase enzyme 2 (COX-2) and its product, prostaglandin E2 (PGE-2). This autocrine loop promotes tumor cell survival (“cell death resistance”) and proliferation under stress [60]. Indeed, COX-2 induction by NETs is a key event that protects HCC cells from cytotoxic stress and apoptosis while enhancing their invasive growth [15,61].

NETs also potentiate angiogenesis and stromal remodeling in HCC [58]. NET-associated MMP-9 degrades the extracellular matrix and liberates sequestered pro-angiogenic factors like vascular endothelial growth factor (VEGF), leading to new blood vessel formation [62]. The result is an inflamed, pro-angiogenic niche that facilitates HCC progression. Notably, NET-induced COX-2/PGE-2 signaling further promotes angiogenesis and immunosuppressive stromal activation [63].

Within these NET “webs”, neutrophil proteases like cathepsin G are released and can trigger the local release of growth factors such as insulin-like growth factor (IGF-1) [64]. IGF-1, in turn, strengthens homotypic tumor cell adhesion via E-cadherin and promotes the formation of multicellular tumor clusters, which enhances the efficiency of metastasis. NET fibers can also tether tumor cells to the endothelium at distant sites, effectively seeding metastatic niches [18,19]. Clinical evidence shows that HCC patients with high NET levels tend to have a more metastatic disease [65].

### 5.1. Effects of NETs on Tumor Growth and Metastasis in HCC

At the same time, NETs actively induce molecular changes in HCC cells that boost their metastatic potential [65]. When NETs trap HCC cells, cancer cells can internalize NET fragments and sense NET-derived DAMPs [3,17]. This triggers TLR signaling in the tumor cells—TLR4 and TLR9—leading to an aggressive inflammatory phenotype [60]. Yang et al. (2020) demonstrated that NET-entangled HCC cells activate TLR4/9 signaling, which upregulates COX-2 and downstream prostaglandin E2, endowing cells with enhanced invasiveness and motility [60]. The NET-TLR4/9-COX-2 axis effectively “licenses” these HCC cells to metastasize, as blocking TLR4/9 or COX-2 erases the pro-metastatic effects of NETs [31]. NET components can also engage cancer cell surface receptors like CCDC25, a recently identified NET-DNA sensor on tumor cells [63]. Through such signaling, NETs push HCC cells toward a more mesenchymal, invasive state, primed for metastasis [66].

Additionally, NETs prepare distant metastatic sites by remodeling the microvasculature and immune environment [67]. NET-associated proteases increase vascular permeability and facilitate the extravasation of tumor cells, while NET-DNA and histones may activate endothelial cells and platelets to form pro-thrombotic scaffolds in target organs [66]. This pro-metastatic niche supports incoming HCC cells. In mouse models, the induction of NETs dramatically increases the incidence of metastases. This may be translated in clinical practice, considering that postoperative inflammation or infection that triggers NETosis leads to greater liver metastasis [26]. Conversely, it was demonstrated that, by degrading NETs with DNase or inhibiting NET formation (PAD4 inhibitors), one can markedly reduce metastatic colonization [26]. Thus, NETs both pave the way for tumor cells to metastasize and push tumor cells to become more aggressive travelers. Targeting these NET-driven processes (e.g., with NET inhibitors or DNase) has been shown to blunt HCC metastasis [65].

### 5.2. NETs and Immunosuppression in HCC

On the other hand, NETs contribute to immune escape in HCC by forming physical barriers and releasing immunosuppressive factors [32]. NETs promote CD73 upregulation and adenosine production via Notch2–NF-κB signaling, inhibit cytotoxic T and NK cells through the TMCO6-TCR pathway, and attract regulatory T cells and MDSCs via chemokines (e.g., CCL2, CXCL8), creating an immunosuppressive TME [68,69]. The TME of HCC is characterized by the accumulation of inflammatory cells, creating a pro-thrombotic microenvironment [70,71]. Yang et al. performed a translational study integrating human HCC tissue analysis, in vitro assays, organoid models, and preclinical mouse experiments, demonstrating that NETs accumulate in immune-rich regions of HCC, including the tumor margins, sites of microvascular invasion, and areas surrounding portal vein tumor thrombi. They further described the mechanism by which NETs mediate immune escape in HCC by upregulating CD73 [70]. In the TME, neutrophils are activated by hypoxemia and other signals, such as granulocyte colony-stimulating factor (G-CSF) and granulocyte-macrophage colony-stimulating factor (GM-CSF), promoting NET release. NETs assist tumor cells by remodeling the environment, promoting invasion, and protecting them from immune attacks [57,58,71]. Clinical data show that elevated NET levels correlate with HCC metastasis, especially in the lungs, due to the unique vascular architecture [60]. The interplay between NET-mediated immune suppression, the TME, and recurrence forms a complex triad requiring further investigation [35,60].

### 5.3. NETs and Epithelial–Mesenchymal Transition

Another recently described key mechanism by which NETosis is implicated in HCC progression is the epithelial–mesenchymal transition (EMT), a dynamic process during which epithelial cells lose their stability and convert into a mesenchymal phenotype, gaining the capacity for migration and metastasis, first into the surrounding tissues and then into the circulation [72].

The EMT process is regulated by signaling pathways (hypoxia via HIF, TGF-β, Notch, growth factors, Wnt), transcription factors (TFs) (SNAIL, ZEB, TWIST, and others), miRNAs, and epigenetic modifications (e.g., DNA methylation) [73,74]. Moreover, NE from NETs can induce EMT and motility by activating the Src-PI3K-Akt pathway in cancer cells [25].

Maddalena et al. reviewed all possible mechanisms of NETs’ implications in EMT [75]. Endothelial cells in contact with NETs lose VE-cadherin, releasing junctional β-catenin [75,76]. As was shown, β-catenin was translocated into the nucleus and caused the upregulation of Snail1 [76]. Moreover, studies revealed a reduction in E-cadherin levels and increased expression of fibronectin and β-catenin [77]. Mechanisms include the loss of cell epithelial characteristics—adhesion and polarity—changes in the cytoskeleton, and the acquisition of mesenchymal characteristics: increased motility and resistance to apoptosis and invasiveness [78,79].

As in other cancers, EMT is significantly involved in HHC development and progression, where tissue heterogeneity is one important characteristic [80]. This involvement has been demonstrated by studies finding EMT markers in HCC specimens in 56% of patients, while newer and more sensitive methods to detect EMT, like the use of circulating tumor cells, detected EMT markers in up to 66% of patients [80]. Moreover, the EMT process may be involved in HCC therapeutics, where using EMT regulators like TGF-β receptor I kinase may inhibit/slow HCC progression. On the other hand, hypoxia developed during transcatheter arterial chemoembolization (TACE) with consecutive EMT activation may partially explain the variable response of HCC to this technique [81].

## 6. How Can We Detect and Monitor NETosis? Is It Clinically Relevant and Reliable?

Over time, direct and indirect methods of assessing NETs in clinical practice have been explored [82,83]. Although NETosis has been associated with cancer progression, its value in defining risk groups remains uncertain [24,82]. Moreover, standardized assessments of sensitivity and specificity for each diagnostic technique are still lacking, and pathology-specific cut-off values have not yet been established [82]. Three methods of assessing NETs are usually utilized in clinical practice: enzyme-linked immunosorbent assays (ELISAs), flow cytometry, and quantitative PCR (qPCR) [82].

ELISA provides a practical and clinically relevant approach to quantifying NETosis in patients with HCC, offering diagnostic and prognostic utility [84,85]. This assay typically detects complexes of DNA bound to neutrophil-specific proteins, such as MPO and NE [86,87,88]. Another NET-associated marker commonly detected by ELISA is citrullinated histone H3 (CitH3). Studies have shown that HCC patients exhibit markedly elevated preoperative MPO-DNA levels compared with healthy controls [89]. Higher circulating MPO-DNA levels correlate with significantly shorter recurrence-free and overall survival in HCC cohorts [89]. Furthermore, NET-associated markers in malignant ascites from HCC patients, such as double-stained DNA, CitH3, MPO, and MPO-DNA, are markedly elevated compared with benign ascites, suggesting the diagnostic role of ELISA in clinical settings [90].

Flow cytometry quantifies NET formation at a cell level. Neutrophils undergoing NETosis lose membrane integrity and release DNA, becoming positive for cell impermeable DNA dyes [84]. Sytox Green-positive neutrophils enable the quantification of the fraction of neutrophils that release NETs [91]. Alternatively, dual-antibody staining can be used (e.g., anti-citrullinated H3 and anti-neutrophil MPO) to identify neutrophils that intracellularly [92,93]. Flow cytometry can rapidly analyze thousands of cells, providing an objective, quantitative measure of NETotic cells [83,94].

qPCR assays quantify NET-associated cell-free DNA (cfDNA) in patient blood, including the separate measurement of nuclear and mitochondrial cfDNA [95]. Complementarily, RT-qPCR can assess the expression of NET-regulating genes such as PAD4, MPO, and NE, offering insight into neutrophil activation and the NET-forming capacity.

## 7. NETosis in Hepatocellular Carcinoma

HCC exemplifies the clinical importance of NETosis in cancer. Patients with HCC show elevated NET activity in both blood and tumor tissue [61]. Tumor-associated neutrophils in HCC are primed for NETosis; studies have found significantly higher NET formation in HCC tumor samples compared to adjacent normal liver tissue [96]. Neutrophils isolated from HCC patients (especially those with metastatic disease) release more NETs than neutrophils from healthy individuals [60]. Clinically, NET biomarkers are emerging as prognostic indicators in HCC. One study reported that HCC (and cholangiocarcinoma) patients had dramatically higher pre-surgery circulating NET levels (measured as MPO-DNA complexes) than healthy controls, and those with high NET levels had significantly shorter recurrence-free and overall survival [89]. High intratumoral NET content (citrullinated histone H3) also predicted early recurrence and poorer survival in these patients [89]. Moreover, NETs may underlie severe HCC complications: for example, circulating NET markers are elevated in HCC patients with portal vein tumor thrombosis, a lethal event that can lead to metastases [43].

By contrast, Zenlander et al., in a clinical study on 227 patients with cirrhosis, HCC, and controls, found that CitH3 and MPO-DNA were significantly elevated in patients with end-stage liver cirrhosis and HCC as compared with controls. They found that the presence of HCC did not further increase the plasma levels of MPO-DNA or CitH3 as compared to Child–Pugh B and C cirrhosis. The levels of MPO-DNA and CitH3 were similar in patients with and without a history of thrombosis [87].

More recently, a genomic analysis also showed a NET-associated prognostic signature in patients with HCC and even identified GAS2L3 as a gene linking NETosis to tumor progression and therapeutic potential [97].

## 8. The Effects of Anesthetic Drugs on NET Formation: What Is the Clinical Evidence?

### 8.1. Propofol vs. Inhalational Anesthesia

In addition to their recognized hypnotic effects, these drug classes also influence neutrophil function by reducing CD11 expression, impairing chemotaxis, diminishing the oxidative burst, and decreasing phagocytic activity. There are only a few studies, summarized in Table 1, on the influence of propofol and inhalation anesthesia on NETosis [98].

Most studies are preclinical, with only a few small randomized controlled trials (RCTs) showing that propofol modulates the inflammatory response and suppresses T-cell function as well as T-cell-dependent immune activity [99,100]. It regulates the role of macrophages in infections, inflammation, and tumors; inhibits the production of pro-inflammatory cytokines (IL-6, TNF-α), and decreases ROS production [101,102,103].

Preclinical studies mostly also show that propofol inhibits neutrophil function or attenuates exaggerated neutrophil activation [104,105]. Meier et al., in a preclinical experimental study using in vitro models with isolated human neutrophils, established that propofol and a lipid emulsion significantly inhibited ROS-dependent NET production [99]. In another preclinical experimental study using in vivo and in vitro murine models, Yamamoto et al. demonstrated that in vitro propofol inhibited T-cell glycolytic activity, effector function, and differentiation, and, in vivo, they demonstrated a suppressive effect of propofol on T-cell-dependent immune responses [106].

The impact of inhalational anesthesia on the immune response in cancer surgery remains unclear [107,108]. While current clinical data, mostly from small studies, do not show significant differences between TIVA and volatile agents, experimental studies suggest that sevoflurane may determine immunosuppressive effects, particularly in animal and in vitro models [109,110]. Thus, Chen et al., in a preclinical study on neutrophils, reported that propofol reduced NET formation by inhibiting p-ERK and hypochlorous acid (HOCl) [111].

Despite these findings, Galos et al. performed a single-center, prospective, double-blinded RCT on 120 patients undergoing primary breast tumor resection. Women were randomly assigned to receive one of four anesthetics: sevoflurane (S), sevoflurane plus i.v. lidocaine (SL), propofol (P), and propofol plus i.v. lidocaine (PL). This study did not find a significant difference in NET formation when analyzing serum MPO-DNA and CitH3 between propofol-based total intravenous anesthesia (TIVA) and inhalational anesthesia in breast cancer [112]. Similarly, in another prospective, randomized, single-blinded clinical trial on 40 patients comparing inhalation anesthesia with propofol-regional anesthesia, also in breast cancer, Aghamelu et al. did not find a significant difference in the serum expression of MPO (10.5 ± 6.6 vs. 11.5 ± 4.7, ng mL^−1^, *p* = 0.60) and CitH3 (3.6 ± 2.3 vs. 4.0 ± 5.9, ng mL^−1^, *p* = 0.80) between these two anesthetic techniques [113].

In addition, Zhang et al. performed a prospective, single-center, controlled, parallel-group RCT on 119 patients undergoing primary or invasive breast tumor resection to receive one of four anesthetics: sevoflurane (S), sevoflurane plus i.v. lidocaine (SL), propofol (P), and propofol plus i.v. lidocaine (PL), analyzing three serum markers of NETosis—CitH3, MPO, and NE—and one for angiogenesis (VEGF-A). They found a within-group difference in preoperative and postoperative marker expression for MPO (S group: 10.39 [6.89–17.22] vs. 14.31 [8.55–20.87] ng mL^−1^, *p *= 0.032; P group: 9.45 [6.73–17.37] vs. 14.34 [9.87–19.75] ng mL^−1^, *p *= 0.035) and NE (S group: 182.70 [85.66–285.85] vs. 226.20 [91.85–391.65] ng mL^−1^, *p *= 0.045; P group: 154.22 [97.31–325.30] vs. 308.66 [132.36–483.57] ng mL^−1^, *p *= 0.037), but they did not find a between-group difference [9].

Taking into consideration that the TME plays an important role in HCC biology, we searched for studies focusing on the comparative effects of propofol vs. volatile anesthesia on the TME in HCC, but none could be found. However, it can be speculated that propofol may reshape the TME via anti-inflammatory and anti-angiogenic effects, the regulation of immunity, and remodeling the extracellular matrix [114].

Further large clinical studies are needed to draw a definitive conclusion.

### 8.2. Intravenous Lidocaine and NET Formation

Lidocaine is the only local anesthetic that can be administered intravenously during surgery as an adjunctive analgesic. Numerous studies have reported that i.v. lidocaine during the perioperative period decreases postoperative pain and perioperative opioid use, has pro-peristaltic gastrointestinal effects, contributes to the resumption of bowel function, and has anti-inflammatory effects [115,116].

Lidocaine’s mechanisms of action responsible for these effects include VGSC blockage (with different sensitivities depending on the channel subtype); the blockage of potassium, calcium, and TRP channels; and the blockage of acetylcholine, glutamate, serotonin, and opioid receptors. The anti-inflammatory effects are produced by the inhibition of neutrophils and their functions and of pro-inflammatory cytokines IL-6, TNF-α, and IL-1β [117].

Recently, the effects of lidocaine on NETosis have been also investigated in a small number of both preclinical and clinical studies. In a prospective RCT, as mentioned above, Galos et al. reported that, when lidocaine was added to either TIVA or inhalation anesthesia, CitH3, MPO, and MMP-3 decreased significantly (109 ± 23 vs. 125 ± 22 ng mL^−1^, *p* = 0.01 for SL and S and 98 ± 14 vs. 130 ± 32 ng mL^−1^, *p* = 0.007, for PL and P, respectively), independently of the anesthetic technique, while MMP-9 and VEGF-A did not differ significantly [112].

Zhang et al., using a similar methodology, did not find a significant difference in values between groups with and without lidocaine regarding CitH3, MMP-9, and VEGF-A [9].

Similarly, Zhang H. et al., in a multicenter, double-blinded, prospective, large RCT on 563 patients undergoing a pancreatectomy for pancreatic cancer, reported that intraoperative i.v. lidocaine reduced intraoperative fentanyl and circulating, but not tumor, NETosis. No differences in OS or DFS were recorded [118].

In another prospective, double-blinded, single-center clinical trial, Ren et al. enrolled 132 patients with lung cancer in four study groups: a placebo group (Group C), a lidocaine group (Group L), a dexmedetomidine group (Group D), and a dexmedetomidine plus lidocaine group (Group LD). MPO, CitH3, MMP-3, MMP-9, and VEGF were significantly reduced in the lidocaine and dexmedetomidine groups, respectively, as compared with controls, as well as IL-6 and TNF-α as markers of inflammation. MPO was decreased in Groups L, D, and LD (−197.08 ± 34.01, −137.37 ± 32.41, and −189.45 ± 33.73 U/mL, *p* < 0.001, respectively); CitH3 was reduced in Groups L, D, and LD (−49.51 ± 9.11, −34.80 ± 10.37, and −51.82 ± 8.98 ng/mL, *p* < 0.001, respectively). Pain and quality of recovery were significantly better in the lidocaine group, and this lasted 24 h after infusion stopped [119].

We can only speculate that, by reducing NETosis, i.v. lidocaine may reduce the incidence of recurrence and improve survival in cancer patients undergoing surgery [120]. The studies published so far are mostly small clinical trials following patients for only 1 year postoperatively or focusing only on NETosis biomarkers. Large RCTs following patients for up to 4 years postoperatively are needed to confirm this hypothesis.

Our study (NCT07207304) will hopefully bring new data on the effects of intravenous lidocaine on NETosis in patients with HCC.

### 8.3. Regional Anesthesia

Emerging clinical evidence indicates that regional anesthesia attenuates NET formation in cancer surgery [4]. As mentioned, Aghamelu’s study compared the effects of propofol/paravertebral blockade vs. sevoflurane anesthesia on NETosis. No difference in NETosis expression was observed, as determined by the concentrations of MPO and CitH3 [113].

**Table 1:** Summary of preclinical and clinical studies evaluating the impacts of anesthetic techniques on NETosis in cancer patients.

Study Design	Cancer Type	Anesthetic Protocol	Number of Participants	NETosis Marker Measured	Effect Observed
Chen et al. (2019) Preclinical [111]	N/A	Propofol, Midazolam, Ketamine, Thiomylol Sodium	N/A	Phorbol myristate acetate (PMA)	Propofol inhibited PMA-induced NET formation
Galos et al. (2020) RCT [112]	Breast Cancer	Sevo ± Lidocaine Propofol ± Lidocaine	120	MPO, CitH3	Lidocaine decreased NETosis formation regardless of GA
Aghamelu et al. (2020) RCT [113]	Breast Cancer	Volatile + Opioids Propofol + PPA	40	MPO, CitH3	No difference
Zhang et al. (2024) RCT [9]	Breast Cancer	Sevo + Lidocaine Propofol + Lidocaine	120	MPO, CitH3, NE	No increase in postoperative serum concentration
Zhang et al. (2022) RCT [118]	Pancreatic Cancer	Intravenous Lidocaine	536	Circulating NETs Tumor-Associated NETs	Intravenous lidocaine did not improve overall DFS
Ren et al. (2023) RCT [119]	Lung Cancer	Intravenous Lidocaine Dexmedetomidine	132	NETs, MMPs, VEGF	Lidocaine and dexmedetomidine reduced production of NETs according to tumor metastasis biomarkers
Wu et al. (2023) RCT [121]	Colorectal Cancer	Propofol–Epidural (PEA) Volatile + Opioids	60	MPO, CitH3, MMP-9	Propofol–PEA reduced NETosis

Another recent single-center randomized trial in colorectal cancer patients compared propofol plus thoracic epidural anesthesia (PEA) and standard general anesthesia without epidural (GA) [121]. Propofol–epidural anesthesia reduced NETosis (as assessed by MPO and CitH3) during colorectal cancer surgery as compared with GA [121]. MPO levels were significantly higher in the GA group compared with the PEA group (28.06 ± 11.3 vs. 20.54 ± 7.29 ng/mL; *p *= 0.004). Similarly, CitH3 levels were also higher in the GA group at 24 h (3.22 ± 0.86 vs. 2.73 ± 0.94 ng/mL; *p *= 0.042). A likely explanation is that regional blockade reduced surgical stress and pain-induced immune activation. By preventing intense catecholamine release and lowering opioid exposure, epidural anesthesia may help to preserve a more balanced immune profile, including neutrophil function [121]. Further studies are also needed to better understand the effects of regional anesthesia on NETosis.

### 8.4. Opioids and NETosis

Specific data on opioids and NETosis in cancer patients are limited, since patients typically receive multimodal analgesia [121]. Taking into consideration preclinical studies, excessive opioid (for example, morphine) use might exacerbate NET formation or at least does not decrease NETosis [122]. Preclinical research on mice supports this hypothesis: in a laboratory model, morphine combined with tumor-derived alarmins (like HMGB1) markedly increased NET release from neutrophils [123]. Opioids may thus inadvertently promote NET-mediated pro-tumor effects. In summary, while not definitively proven to increase NETosis in cancer patients, opioids are a plausible contributor to an immunosuppressive and pro-NET environment. Techniques that reduce opioid requirements (like epidurals or IV lidocaine) have been associated with lower NET levels and less immune perturbation in the perioperative period [121].

### 8.5. Dexmedetomdine and α2 Agonists

**Figure 1:**
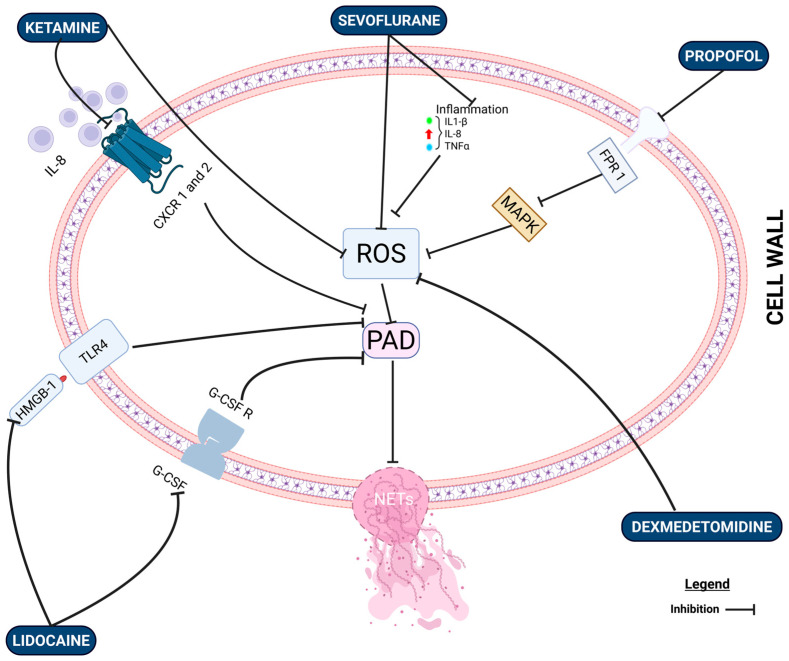
Neutrophils, NETs, and anesthesia. Propofol exerts inhibitory effects. It blocks formyl peptide receptor-1 (FPR1) on the neutrophil surface and inhibits the ERK/MAPK pathway, leading to reduced downstream MPO-derived HOCl production [107,111]. Sevoflurane attenuates neutrophil activation by blocking chemokine receptor 2 (CXCR2) (IL-8 receptor) and reducing β_2_-integrin (CD11b) expression, resulting in less neutrophil adhesion/recruitment and a smaller oxidative burst (lower ROS output) [96]. Lidocaine, administered intravenously, decreases ROS generation, PAD4 activation, and histone H3 citrullination [115]. Dexmedetomidine inhibits TLR2/NF-κB/NLRP3 pathway activation and the subsequent production of ROS [119]. Ketamine reduces ROS levels in a nonspecific and not yet fully understood manner [98]. It also decreases IL-8 expression, thereby contributing to both the direct and indirect inhibition of PAD activity [98,127]. Created in BioRender. S.S. (2025) https://BioRender.com/3ip8gq5.

Although not a new agent in medical practice, dexmedetomidine, a selective adrenergic receptor agonist, is currently widely employed in anesthetic practice for its sedative and analgesic properties, as well as for its putative anti-inflammatory effects, as demonstrated in preclinical models [124]. Its immunomodulatory function has been explained by the release of norepinephrine through the activation of the α2 receptor located on the presynaptic membrane [125]. It has been observed that dexmedetomidine may modulate innate immunity (through the regulation of antigen-presenting cells and other immune cells), as well as adaptive immunity (through T cells), thereby contributing to balanced immune function [125,126]. It may also reduce the levels of pro-inflammatory cytokines (IL-1β, IL-6, IL-8, IL-12/23 [p40], IL-17A, IL-18, IFN-γ, TNF-α, HMGB1, MIP-2, MCP-1) while enhancing the levels of anti-inflammatory cytokines (IL-2, IL-4, IL-10, and TGF-β1) [125]. Corriden et al. hypothesized that dexmedetomidine, through its anti-inflammatory effects, might also inhibit NETosis; however, they concluded that dexmedetomidine, at therapeutically relevant concentrations or even higher, did not directly inhibit NET production by human neutrophils in response to NETosis inducers [124]. Different results were reported in Ren’s study, where the infusion of lidocaine and dexmedetomidine reduced NET production regardless of the intraoperative propofol dose in patients with lung cancer [119]. The major mechanisms by which these anesthetic agents impact NETosis are illustrated in Figure 1.

### 8.6. Limitations of Current Studies and Future Research

As can be seen, most of the studies focusing on the effects of anesthetic techniques on NETosis are small clinical trials investigating the effects on NETosis markers exclusively or adding OS and DFS at 1 year postoperatively. Drugs like i.v. lidocaine or dexmedetomidine require further investigation considering the preliminary positive findings. Across the included studies, post-intervention blood samples used to assess NETosis were collected at different time points, and the administered lidocaine doses also varied. Whether different opioids or doses have the same effects on NETosis also requires investigation.

The analysis of the risk of bias for the included clinical trials, performed using the Cochrane RoB 2 guidelines, showed an overall low risk of bias, with the exception of one study that raised some concerns regarding randomization due to unblinded clinicians [128].

On the other hand, it would be very difficult to separate the effects on NETosis from the effects of anesthetic techniques on immunity/immunomodulation or on cancer cell biology since these exist in a complex network.

Large RCTs are also necessary to investigate the effects of different anesthetic techniques on long-term outcomes in cancer patients undergoing surgery and to correlate the effects on NETosis with long-term outcomes in these patients.

## 9. Therapeutic and Clinical Implications

Growing evidence from human studies indicates that anesthetic management may influence NET formation and related immune biomarkers during cancer surgery [121].

This may have several important implications. Firstly, there may be potential to reduce the metastatic risk [129]. Since NETs are implicated in cancer metastasis and recurrence, strategies that attenuate NETosis might improve oncologic outcomes [121]. Choosing anesthetic techniques that limit NET release—for instance, adding a lidocaine infusion or using regional anesthesia—could theoretically reduce the “pro-metastatic” milieu in the immediate postoperative period. Preliminary reports so far indicate that patients receiving lidocaine or epidurals showed lower levels of NET markers and of other pro-tumor factors after surgery [9,119].

Multimodal analgesia vs. opioid-based anesthesia is another potential implication of NETosis. The findings on analgesia and the effects on NETosis reinforce a shift toward multimodal analgesia in cancer surgery. Incorporating non-opioid adjuncts (lidocaine infusion, regional blocks, acetaminophen, etc.) can achieve analgesia with less opioids. The benefit is twofold: better pain scores and possibly a reduction in NETs and inflammatory cytokines [5,7].

While surrogate markers like MPO and CitH3 are promising indicators for extended cancer metastasis and worse prognoses, the absence of a recognized methodology and a standardized technique with established sensitivity and specificity makes it difficult to use NETosis in diagnosis and outcome monitoring [82]. A potential solution involves using a combination of well-established biomarkers such as alpha fetoprotein (αFP), AFP L3, and des-gamma-carboxy prothrombin (DCP) within diagnostic panels, together with emerging markers including circulating tumor DNA (ctDNA), genomic glycosylation profiles, salivary metabolomics, and microRNAs [130]. This integrated approach may support earlier diagnosis and enable more detailed and personalized follow-up in patients with hepatocellular carcinoma.

Ongoing and future trials are needed to determine if these immunological improvements (lower NETs, preserved immune cells) translate into quantifiable clinical benefits (less metastasis, improved survival) [121]. If confirmed, the anesthetic choice could become a modifiable factor in cancer care protocols.

Human studies show that anesthetic drugs may influence NET formation. Propofol-based or regional techniques tend to be more “NET-sparing” (although not significantly in some studies) compared to inhalational anesthesia with opioids, especially when combined with i.v. lidocaine or neuraxial blocks. As shown before, these approaches reduce circulating NET biomarkers like MPO-DNA complexes and CitH3 after surgery, reflecting a less pro-inflammatory, less pro-metastatic state in the patient. Such immune-modulating anesthetic choices hold promise in improving cancer outcomes and represent an active area of research in perioperative oncology [112,121].

Future studies should focus on the effects of anesthetic drugs on NETosis in HCC patients and their short- and long-term outcomes. The potential impact of adding intravenous lidocaine on the outcomes after TACE also requires further evaluation in large RCTs.

## 10. Conclusions

In conclusion, NETosis is a process that is essentially implicated in HCC biology. The clinical significance of NETosis includes, at present, the possibility of disease staging and the evaluation of prognosis in HCC by evaluating NETosis markers. Preliminary data indicate that certain anesthetic drugs like lidocaine may have a role in decreasing NETosis. Future studies are needed to exactly quantify these effects. Targeting NETs may be another way to influence HCC outcomes.

## Data Availability

No new data were created or analyzed in this study.

## Abbreviations

The following abbreviations are used in this manuscript:

NETs	neutrophil extracellular traps
DNA	deoxyribonucleic acid
HCC	hepatocellular carcinoma
CitH3	citrullinated histone
MPO-DNA	myeloperoxidase–DNA complex
MMP-9	matrix metalloproteinase-9
VEGF	vascular endothelial growth factor
HBV	hepatitis B virus
NAFLD	nonalcoholic fatty liver disease
HBx	HBV X protein
HCV	hepatitis C virus
DAMPs	damage-associated molecular patterns
PAMPs	pathogen-associated molecular patterns
TLR	Toll-like receptor
TNF-α	tumor necrosis factor
pSTAT3	phosphorylated signal transducer and activator of transcription-3
pERK1/2	extracellular signal-regulated kinase 1/2
SYK	spleen-associated tyrosine kinase
TME	tumor microenvironment
TAN N2	tumor-associated neutrophils N2
ROS	reactive oxygen species
IL-8	interleukin-8
IL-1β	interleukin-1β
GM-CSF	granulocyte–macrophage colony-stimulating factor
IFN-γ	interferon gamma
IL-17A	interleukin-17a
IL-6	interleukin-6
i.v.	intravenous
CCL	chemokine ligand
CXCL	chemokine ligand
NADPH	nicotinamide adenine dinucleotide phosphate
PMA	phorbol myristate acetate
DEK	chromatin-binding protein
CDK6	cyclin-dependent kinase 6
PAD4	peptidyl arginine deaminase 4
GSDMD	protein gasdermin D
HMGB1	damage-associated molecular pattern
RAGE	receptor of advanced glycation end-products
COX-2	cyclooxygenase-2
PGE-2	prostaglandin e_2_
TGF-β	transforming growth factor beta
PDGF	platelet-derived growth factor
EGF	epidermal growth factor
IGF-1	insulin-like growth factor 1
CCDC25	coiled-coil domain-containing protein 25
EMT	epithelial–mesenchymal transition
Src	Src family kinase
NK	natural killer
Tregs	regulatory T cells
MDSCs	myeloid-derived suppressor cells
CD73	ecto-5′-nucleotidase
Notch2	neurogenic locus notch homolog protein 2
NF-κB	nuclear factor kappa B
A2A	adenosine a2a
TMCO6	transmembrane and coiled-coil domain 6
TCR	T-cell receptor
G-CSF	granulocyte colony-stimulating factor
ELISA	enzyme-linked immunosorbent assay
PCR	polymerase chain reaction
qPCR	standard quantitative PCR
RT-qPCR	reverse transcription PCR
cfDNA	cell-free DNA
ELANE	neutrophil elastase
DNA-ase	deoxyribonuclease
TIVA	total intravenous anesthesia
RCT	randomized controlled trial
VEGF-A	vascular endothelial growth factor A
GA	general anesthesia
PAF-R	platelet-activating factor receptor
HOCl	hypochlorous acid
TACE	transcatheter arterial chemoembolization
